# Encapsulated Papillary Carcinoma in a Deep Inferior Epigastric Perforator Flap Breast Reconstruction: A Case Report

**DOI:** 10.7759/cureus.35963

**Published:** 2023-03-09

**Authors:** Amos Nepacina Liew, Lavanya Palanimalai, Cyril Tsan

**Affiliations:** 1 Department of Breast Surgery, Monash Health, Moorabbin, AUS

**Keywords:** autologous flap, breast cancer, intracystic papillary carcinoma, diep, encapsulated papillary carcinoma

## Abstract

We report a case of a 45-year-old female who developed multi-focal encapsulated papillary carcinoma (EPC) in her left breast. This was on a background of previous left breast high-grade ductal carcinoma in situ, for which she had a skin-sparing mastectomy and deep inferior epigastric perforator (DIEP) reconstruction five years ago. Papillary carcinoma is a rare pathology, and its occurrence in autologous breast reconstruction is even rarer. This is the second reported case of papillary carcinoma in a DIEP reconstruction. Although surgery remains the gold standard for EPC, debate remains with regard to adjuvant endocrine therapy and radiotherapy. We discuss the diagnosis and current management of an EPC.

## Introduction

Papillary carcinoma of the breast is a rare pathology with an annual incidence of 0.5% of all newly diagnosed breast cancer, a subset of which is classified as encapsulated papillary carcinoma (EPC) [1]. Furthermore, breast cancer recurrence in autologous flaps has an annual incidence of 1% [2]. Hence, papillary carcinoma in an autologous flap is a rare event. Patients normally present with a slow-growing mass, with post-menopausal women experiencing bloody discharge. Although surgical excision is the gold standard, there remain questions regarding the usage of adjuvant endocrine therapy or radiotherapy, given its largely indolent nature [3]. Hence, we aim to summarize the currently available literature on the management of an EPC.

## Case presentation

A 45-year-old female was referred to our tertiary centre outpatient breast surgery clinic with multifocal left-sided breast lesions in a previously reconstructed deep inferior epigastric perforator (DIEP) flap. This was on a background of previous left breast high-grade ductal carcinoma in situ (DCIS), oestrogen and progesterone positive, with no nodal metastasis which was treated with a left skin-sparing mastectomy and DIEP reconstruction in 2016 and was commenced on adjuvant tamoxifen, which was ceased in 2021. She subsequently relocated overseas.

**Figure 1:**
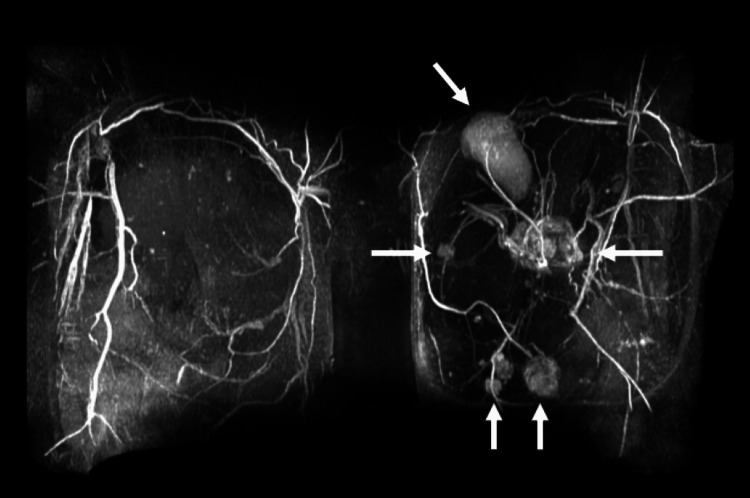
3D-reconstruct of MRI breast showing multifocal left-sided breast lesions (antero-posterior view). MRI: magnetic resonance imaging

Earlier this year, she noticed a palpable lump at 6 o’clock (oc) 6cm in the reconstructed left breast while off tamoxifen. A magnetic resonance imaging (MRI) of the breast pursued overseas showed four lesions that were core biopsied (Table 1). The patient decided to return to Australia for further management. Given that the MRI images could not be attained from overseas, a repeat MRI was conducted at our institution which revealed one more lesion (Figure 1/Table 1). All imaged pathological lesions were core biopsied.

**Table 1:** Findings of overseas MRI and additional findings from repeat MRI. MRI: magnetic resonance imaging

Position (Left)	Size	Characteristics	Initial histopathology/Cytology
MRI done overseas			
11oc, 4cm	1.9cm x 2.2cm	Fluid-filled lesion	Benign
6oc, 6cm	1.6cm x 1.4cm	Lobulated mass	Encysted Papillary Carcinoma
6oc, 7cm	1.4cm x 1.4cm	Lobulated mass	Encysted Papillary Carcinoma
Retroareolar	3.5cm x 3cm	Peripheral irregular enhancement with nodular wall thickening	Encysted Papillary Carcinoma
Repeat MRI done at our institution (Further findings)			
9oc,13cm	1.2cm x 1.0cm	Lobulated mass	Encysted Papillary Carcinoma

**Figure 2:**
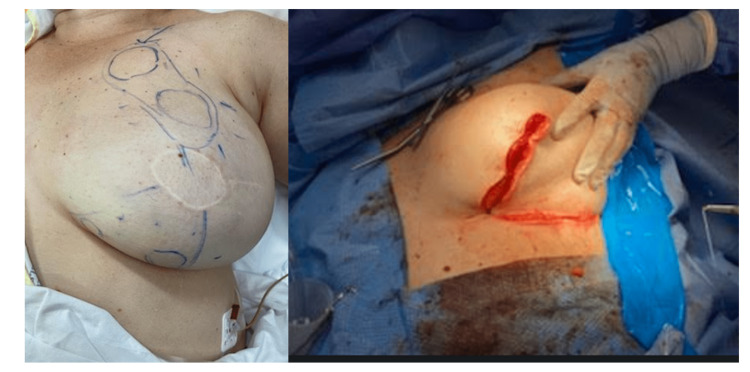
(Left) Pre-operative markings of breast lesions with an inferior wedge mammoplasty approach. (Right) Post-operative closure of inferior wedge mammoplasty.

Following discussion at our breast reconstruction multidisciplinary meeting, the decision was made to pursue wide local excisions of all the lesions. This was done through an inferior wedge mammoplasty at the 6oc position to incorporate two lesions. From that position, the DIEP flap was carefully undermined to prevent injuring the tributary arteries to get to the 9oc, 11oc and retroareolar lesions (Figure 2).

The histopathology showed that these lesions contained epithelial proliferation within forming sheets, cribriform and fibrovascular core structures. They lacked a myoepithelial layer staining within the lesion and the periphery with overall features of an EPC with no infiltration, estrogen receptor (ER) and progesterone receptor (PR) positive. Furthermore, there were moderate nuclear variation and occasional mitotic figures. There were several positive margins at the 11oc and 9oc regions and a re-excision was conducted which subsequently revealed, two foci of intermediate-grade DCIS but clear margins at these sites. She received adjuvant radiotherapy and tamoxifen, and six monthly follow-ups with imaging were proposed.

The patient had a routine review at our post-operative clinic. Initially, her post-operative recovery was hindered by wound complications that subsequently healed with regular dressing changes.

## Discussion

Breast cancer recurrences in an autologous flap are rare and have an annual incidence of 1% [2]. Several possible reasons include circulating tumour cells with local metastasis to the flap prior to surgery, tumour cells seeding at the time of surgery, or lack of microscopic clearance at the mastectomy operative bed [4]. However, there has only been one reported case of a papillary carcinoma recurring in a DIEP flap from the literature review [2].

Papillary carcinoma has an annual incidence of only 0.5% of all newly diagnosed breast cancer [1]. They comprise a heterogeneous group of lesions, which includes EPC with or without invasion. EPC is characterised by a lack of myoepithelial cells in the papillae and is usually ER and PR positive, and lacks HER2 amplification [5]. They can generally be staged and managed as DCIS according to the World Health Organisation (WHO) Classification of Tumours of the Breast (2012) [6]. However, in tumours with high nuclear-grade features and high mitotic activity, they should be managed as invasive breast cancer [5]. Patients commonly present with a slow-growing palpable mass, and post-menopausal women can experience bloody discharge [5]. EPC has an overall good prognosis, with a survival rate of 100% at 10 years [3]. Axillary metastasis remains extremely rare, and the decision to pursue axillary staging remains an area of contention [7].

Surgical excision remains the gold standard for EPC. This is recommended if the core biopsy shows atypia or invasion and in all cases when a solid mass in the cyst is seen [7]. Our team decided to pursue breast-conserving surgery (BCS) for our patient’s disease with no sentinel node biopsy given the largely indolent nature of EPC and negative imaging for axillary metastasis. It is recommended that a 2mm resection margin is achieved when undergoing BCS [8]. Although the majority of EPC are ER and PR positive, HER2 is not amplified, and evidence is still lacking with regard to adjuvant endocrine therapy, mainly with tamoxifen [7]. This is mainly indicated for patients whose tumours appear repeatedly, or age lower than 50 years old [6]. Further research is required to delineate the efficacy of adjuvant radiotherapy in EPC. However, it is recommended that patients with associated DCIS or micro invasion receive a course of adjuvant radiotherapy [9].

Furthermore, the ideal thickness of the skin flap raised during mastectomy is an area of contention. There are varying guidelines with regard to mastectomy flap thickness. A literature review of this topic had varying results with some researchers recommending a flap thickness of around 5mm [10], while others stated that anything less than 8mm has a high risk of ischaemic complications [11]. There are multiple factors including the patient's body habitus and volume of pre-operative breast tissue that has to be factored in when performing a therapeutic mastectomy and raising a skin flap [12]. Hence, this remains an area where further research is required.

## Conclusions

We present a rare case of EPC in a DIEP flap. There are several areas that could be explored in future research with regard to EPC, including sentinel node biopsies with BCS and the efficacy of adjuvant endocrine therapy in overall survival rates for patients with associated DCIS or micro invasion. Nonetheless, EPC generally offers a good prognosis and early detection and resection remain the gold standard treatment.
